# A Scoping Review on Salivary Oxytocin and Vasopressin Measurement in the Dog

**DOI:** 10.3390/ani15162421

**Published:** 2025-08-18

**Authors:** Marta De Santis, Margherita Soncin, Valentina Bertazzo, Luca Martelli, Diletta Fornasiero, Franco Mutinelli, Laura Contalbrigo

**Affiliations:** 1 Italian National Reference Centre for Animal Assisted Interventions, Istituto Zooprofilattico Sperimentale delle Venezie, Viale dell’Università 10, 35020 Legnaro, Italy; fmutinelli@izsvenezie.it (F.M.); lcontalbrigo@izsvenezie.it (L.C.); 2 Laboratory Medicine Service, Istituto Zooprofilattico Sperimentale delle Venezie, Viale dell’Università 10, 35020 Legnaro, Italy; vbertazzo@izsvenezie.it; 3 Epidemiology and Risk Analysis in Public Health, Istituto Zooprofilattico Sperimentale delle Venezie, Viale dell’Università 10, 35020 Legnaro, Italy; lmartelli@izsvenezie.it (L.M.); dfornasiero@izsvenezie.it (D.F.)

**Keywords:** salivary oxytocin, salivary vasopressin, dog, canine saliva, dog welfare

## Abstract

Oxytocin (OT) and vasopressin (AVP) are attracting interest as potential measures for dog welfare and human-dog interactions. The potential for detection and measurement of these two neuropeptides in canine saliva provides a viable alternative to more invasive collection methods. This scoping review aims to analyse the scientific literature measuring salivary OT and AVP in dogs, the methods employed, and the areas of research. A comprehensive search was conducted on the Web of Science, Scopus, and ProQuest platforms, yielding a selection of peer-reviewed, primary research studies that specifically measured dog salivary OT and/or AVP. The final selection comprised 16 articles from 2017 to 2024, offering insights into the methods employed for the collection, processing, and analysis of dog saliva samples. The findings highlighted significant variability in OT and AVP salivary concentrations, which also depended on the methods employed. The research areas of the included articles were primarily linked to the social and maternal behaviour spheres. This study draws from extant research to present an overview of the challenges associated with measuring salivary OT and AVP in dogs. It provides a comprehensive picture of this multifaceted issue, which is crucial for accurately interpreting these peptides as indicators of dog welfare.

## Simple Summary

Salivary hormones have been identified as a potential solution to the challenge of measuring animal welfare non-invasively. In mammals, the neuropeptides vasopressin and oxytocin have a variety of functions and have been associated with social bonding. The potential of oxytocin to serve as an indicator of a positive welfare state has also been suggested in various animal species, including the domestic dog. The present study aims to identify and describe scientific literature pertaining to the measurement of oxytocin and vasopressin in canine saliva. The objective of this review is twofold: firstly, to establish the current state of the art in this field; and secondly, to identify the challenges and potentialities associated with the collection of these hormones from saliva.

## 1. Introduction

A major challenge in the field of animal welfare science is the identification of reliable indicators of the affective states of animals, given the inherent difficulty of verbally questioning them about their welfare [1,2,3]. The analysis of physiological indicators that may reveal positive or negative subjective experiences can offer valuable insights into an animal’s internal processes [3]. A series of messengers, including hormones and neurotransmitters, are typically released in response to specific stimuli to elicit the most appropriate adaptive response from the body. Consequently, these molecules can serve as useful indicators in animals, particularly when their collection can be achieved through relatively non-invasive methods, such as salivary sampling, and subsequent analysis can be accomplished through low-cost and rapid techniques [4,5]. Indeed, as a component of the body fluid heritage, saliva offers the possibility of measuring hormonal concentrations, constituting a possible alternative to blood withdrawal in many species, including dogs [6,7].

In dogs, salivary cortisol levels have already been utilised as a biomarker of the Hypothalamic-Pituitary-Adrenal (HPA) axis, involved in the stress response, in a number of studies [8,9]. However, given the complexity of the physiological systems involved in the body’s attempt to maintain homeostasis, recently other analytes and the use of combined measures are attracting attention for their potential applications in dog welfare assessment [10]. Among these, the peptides oxytocin (OT) and vasopressin (AVP) are of particular interest for several reasons. First of all, these two molecules are related and intertwined as part of the oxytocin-vasopressin system [11], and they interact with the HPA axis in the physiological stress response [11,12]. Thus, they could prove more informative than cortisol alone. Secondly, both hormones are involved in cognition and social behaviour [13], and OT has been studied at the interspecific level as an affiliative indicator released during human-dog interactions [14,15]. Finally, the effects of the two peptides are, in certain instances, analogous, and in others, antagonistic. For example, AVP appears to contribute to the activity of the sympathoadrenal axis involved in the stress response and to the genesis of anxiety and aggressive behaviour, while OT has been implicated in the establishment of bonds, parental behaviour, and the inhibition of the sympathoadrenal activity [16]. Consequently, despite the presence of discrepant findings and the complex interplay between the two peptides and other biological factors, which complicates their interpretation, OT is increasingly being considered as a possible indicator of positive emotional valence in animal welfare science [4,17,18,19,20]. This paves the way for the challenging yet crucial endeavour of identifying positive welfare indicators that could potentially assist in transcending the notion that animal welfare can only be evaluated by the absence of negative states [21,22].

Given the potential relevance of these two peptides for research in the fields of animal welfare and human-animal interaction, and the relatively recent development and validation of assays to analyse them in canine saliva samples, the aim of this scoping review is to examine the scientific literature that has analysed salivary OT (sOT) and AVP (sAVP) in the dog, the methodologies employed, and the challenges encountered.

## 2. Materials and Methods

### 2.1. Search Strategy and Selection of the Sources of Evidence

The present scoping review was conducted following the Preferred Reporting Items for Systematic Reviews and Meta-Analyses extension for Scoping Reviews (PRISMA-ScR) [23]. The review encompassed studies pertaining to OT and AVP measurements derived from dog saliva. No geographical or date restrictions were applied to the selection of studies. The final selection comprised peer-reviewed studies reporting primary data in English, excluding conference proceedings. This choice was made to limit the scope of the review due to time and resource limitations. Qualitative studies, reviews, books, commentaries, editorials, and letters were excluded in accordance with the objective of the review. The literature search was conducted in September 2024 through Web of Science (WoS), Scopus, and ProQuest. The search query was: (dog* OR canine* OR pup*) AND (oxytocin OR vasopressin) AND saliva*. Following the removal of duplicates, the initial screening of titles and abstracts was conducted by a single reviewer (M.D.S.). The full-text screening was then carried out independently by two reviewers (M.D.S. and L.C.). Any disagreement was resolved through discussion or confrontation. Citationchaser, an online tool developed for backward and forward citation chasing [24], was then used to retrieve additional resources from the full-text papers selected for inclusion. The list of references and citations was downloaded (in December 2024) and screened to retrieve any additional source of information by applying the same eligibility criteria described above.

### 2.2. Data Charting and Synthesis

The full-text articles that were included in the study were charted iteratively, with the systematic review by Cobb and colleagues [8] on dogs’ salivary cortisol serving as a model. Initially, articles were grouped according to whether they investigated both sOT and sAVP or solely sOT or sAVP. Furthermore, given the presence of articles that were focused on the development and validation of sOT/sAVP analysis methods, they were considered separately from studies focused on data collection. The following sections and items were included in the data charting: (a) characteristics of the studies (year of publication, Journal, country); (b) objective of the study; (c) methods for salivary collection (device used, use of stimuli for salivation, duration of collection); (d) characteristics of the sample (sample size, breed, age, dog type); (e) aspects related to the experimental design (groups, conditions, timepoints); (f) sample processing and analysis method; (g) concentrations of the analyte (sOT/sAVP); (h) other measures collected. With regard to the age of the sample in each study, for the purpose of synthesis, the age range (or the mean age when the range was not reported) was used to derive age classes on the basis of the article by Harvey [25]. Furthermore, the concentrations of hormones were converted to picograms per millilitre (pg/mL) when reported in other units (nmol/L). Extracted data have been summarised as numbers and/or percentages and are presented in tables or graphs, depending on the most effective graphical visualisation, according to the authors. These tables and graphs are accompanied by a summary of the aims and main results of the articles included.

## 3. Results

### 3.1. Selection of Studies

**Figure 1:**
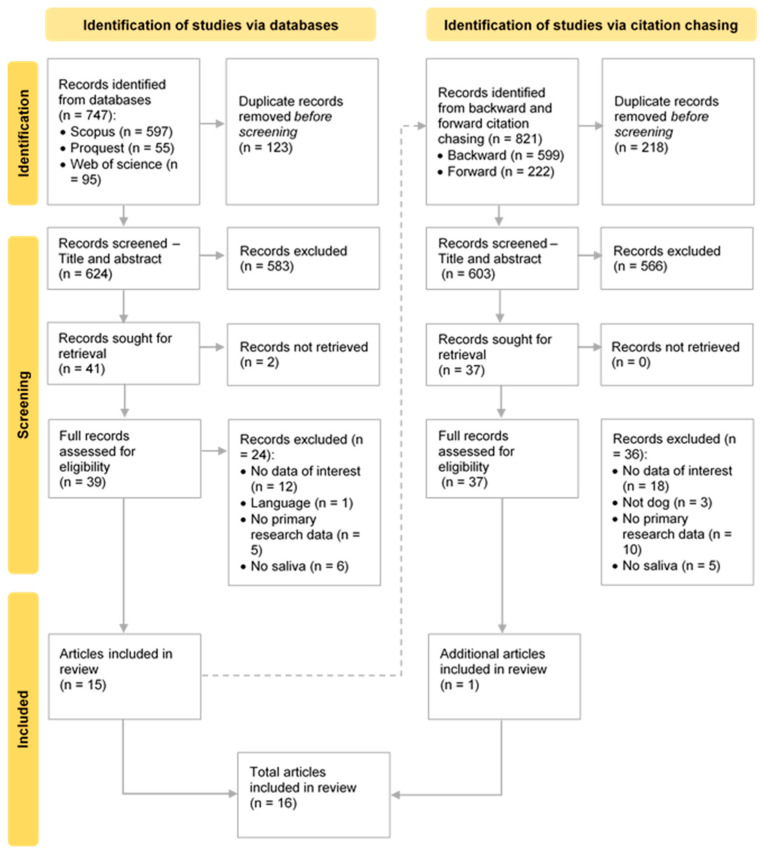
PRISMA flow diagram showing the study selection process. Adapted from Page et al. [26].

The process of study selection is illustrated in the PRISMA diagram in Figure 1 [26]. The first screening of 747 records resulted in the inclusion of 15 articles. A subsequent screening of the citations and references of these 15 articles was conducted to identify additional articles for inclusion in the review, leading to the inclusion of an additional article, bringing the total to 16 articles.

### 3.2. General Characteristics of the Included Studies

Of the 16 included studies, three investigated both salivary oxytocin and vasopressin, 11 investigated only oxytocin, and two investigated only vasopressin. Furthermore, three of the 16 studies were specifically aimed at developing and/or validating methods for the analysis of salivary oxytocin [27,28,29] and vasopressin [27]. The objectives and main findings of these latter studies are presented in Section 3.3.

**Figure 2:**
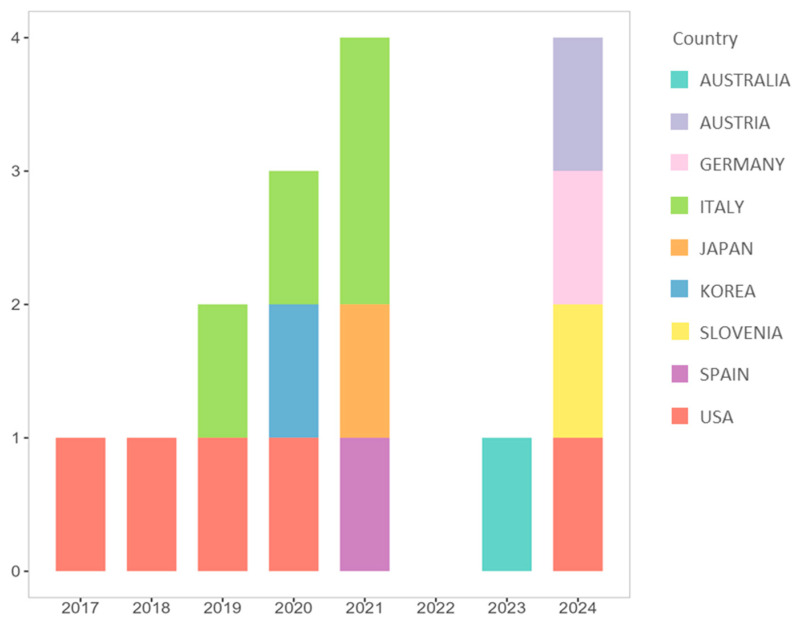
Number of articles reporting data on salivary oxytocin/vasopressin in dogs published each year with countries of publication (n = 16).

Regarding the publication characteristics of the 16 included studies, as shown in Figure 2, the articles were published in the last decade (from 2017 onwards), with an upward trend, except for the years 2022 and 2023, when there was a decline in production. The studies were conducted in various countries, with the majority being conducted in the USA (n = 5) and Italy (n = 4). A total of eight out of the sixteen studies were published in the Journal Animals (MDPI).

### 3.3. Development and Validation Studies for Salivary Oxytocin (sOT) and Salivary Vasopressin (sAVP)

The three studies conducted to validate analytical methods for the analysis of sOT and/or sAVP in dogs employed different collection, processing, and analytical methods. The detailed data charting for each study can be consulted in the Supplementary Material, comprising sOT and sAVP concentrations values when reported (see Supplementary Table S1 for sOT and Supplementary Table S2 for sAVP), while a summary of the diverse methodological approaches of these three development and validation studies is presented in Table 1.

MacLean and colleagues [27] conducted a study with the twofold objective of methodological validation of sOT and sAVP analysis and biological validation of sOT as compared to plasmatic OT in association with nursing. The methodological validation tested, in synthesis: (i) different methods for saliva collection (kind of synthetic swab and salivation stimulation); (ii) different processing procedures (extraction and non-extraction); and (iii) different kits for analysis of sOT and sVP, based on ELISA or high-performance liquid chromatography–mass spectrometry (HPLC-MS). This study was the first and unique in its comparison of collection methods using different synthetic swabs and salivary stimulation techniques, which revealed variability in results depending on the method used. In terms of sample processing, extraction affected both sOT and sAVP concentrations, and in the case of sAVP, extracted and non-extracted samples did not correlate. Regarding the analytical methods, the concentrations of sOT and sAVP detected by ELISA (especially in non-extracted samples) were much higher than those detected by HPLC-MS and also higher than those found in humans. The biological validation experiment confirmed an increase in both salivary and plasmatic OT during nursing, but this increase was significant only for sOT.

The remaining two studies of this section pertain specifically to sOT analytical methods. The study by Wang and colleagues [28] was aimed at developing and validating a liquid chromatography-mass spectrometry (LC-MS) method for dog sOT analysis. Following an initial phase of analytical validation, LC-MS was utilised for OT measurement in dog saliva samples, yielding sOT concentrations that were within the same order of magnitude as those reported by the above-mentioned study by MacLean and colleagues [27]. Finally, the study by López-Arjona and colleagues [29] aimed to evaluate and validate monoclonal and polyclonal assays (AlphaLISA) for measuring dog sOT. This study also reported variations in hormonal concentrations according to differences in both sample processing (reduction/alkylation of the sample) and the analytical methods. In particular, higher oxytocin concentrations (ng/mL versus pg/mL) were found using the polyclonal assay than when using the monoclonal assay or the ELISA kit. Following the validation process, the authors proceeded to conduct an experimental trial, in which dogs were exposed to stroking by their owners, and samples were analysed using both monoclonal and polyclonal assays. This segment of the study will be addressed in Section 3.4.

### 3.4. Studies Measuring Salivary Oxytocin (sOT) in Dogs

A total of 12 studies reported measures of sOT in canines, aside from the findings reported in Section 3.3. Of these, two studies [30,31] also measured sAVP. The detailed data charting for each study can be consulted in the Supplementary Material, comprising sOT concentration values when reported (see Supplementary Table S3).

The following five groups of studies were identified on the basis of their aims: human-animal interaction (HAI) [29,30,31,32,33]; animal-assisted services/interventions (AAIs) [34,35]; dog walking [36]; dog’s cooperation or social cognition [37,38]; and lactation [39,40]. These groups, rather than being derived from a preconceived conceptual framework, were inductively derived from the objectives of the studies during data charting and are useful for the purpose of synthesis.

The five studies measuring sOT in relation to HAI focused on exposing dogs to interactions with humans in order to analyse their effect on behavioural and hormonal parameters, sOT. These interactions were controlled to elicit positive emotions (e.g., petting, stroking, gentle speech, eye contact, play) [29,30,32] and/or negative emotions (e.g., separation and isolation) [31,32]. Alternatively, interactions could be naturalistic, as in the study by Gnanadesikan and colleagues [33], who sought to explore both human and canine behavioural and hormonal responses in familiar and unfamiliar dog-child dyads. To summarise the findings of this group of studies, with a particular focus on sOT, positive 5–10 min HAI resulted in a significant increase in sOT in all studies exposing dogs to controlled positive conditions [29,30,32]. This increase was observed in dogs interacting with both familiar [29,32] and unfamiliar individuals [30]. However, in the study by López-Arjona and colleagues [29], two groups of dogs were compared according to their behaviour during stroking and their acceptance of the salivary collection procedure. A significant decrease in sOT was observed in the group of dogs showing reluctance to accept the sponge for salivary collection and/or not displaying signs of relaxation during stroking. On the other hand, negative HAI (isolation or separation from the owner) did not lead to a significant variation in sOT in the analysed studies [31,32]. Finally, in the study on dog-child natural interactions [33], dogs’ sOT concentrations increased after 15 min of interaction with familiar children (in pet dogs) while decreasing after the interaction with unfamiliar children, although this effect was not statistically credible.

Two other studies included in this review focus on the welfare of therapy dogs during AAIs. These studies are characterised by repeated measurements of multiple physiological parameters that can be indicative of the dog’s emotional state, including sOT. Specifically, Clark and colleagues [34] investigated the impact on therapy dogs of 20 min individual animal-assisted activity (AAA) sessions with people affected by fibromyalgia. The researchers collected dog saliva, tympanic membrane temperatures, and cardiac parameters. In contrast, the single case study by Hill and colleagues [35], compared behaviour and salivary biomarkers (cortisol, OT, and alpha-amylase) in a single dog when resting or when involved in 50 min animal-assisted occupational therapy sessions with children diagnosed with autism spectrum disorders, across 7 weeks. In both cases, no statistically significant differences in sOT were identified (and similarly for the other salivary biomarkers analysed), suggesting minimal impact on the performed AAIs on the dog’s welfare.

A distinct investigation was undertaken to examine the impact of 30 min of dog walking on both owners and their dogs [36]. The study involved the analysis of salivary OT and cortisol, as well as brain neural activity (through monoamines and GABA). However, the authors did not report any variation in dogs’ sOT levels taken at four timepoints (before, after 15 and 30 min of walking, and 10 min after the end of the walking session), nor for the dogs’ other measurements. The mean sOT values for dogs were found to be comparable to those reported in other studies, and the dogs’ sOT levels were found to be approximately four times higher than those observed in their owners.

Two further studies examined cooperation and social cognition. Specifically, the study by McGetrick and colleagues [37] sought to ascertain whether changes in sOT concentration were associated with the experience of a cooperative or non-cooperative act and could be predictive of reciprocation in dogs through an experiment with familiar and unfamiliar dogs. However, the results indicated that sOT concentrations remained unaffected by the varying testing conditions. The second study utilised a two-way object test to examine the relationship between a dog’s cognitive performance, the dog-owner relationship, and sOT concentration in French Bulldogs [38]. No association was identified between sOT, dog behaviour during testing, and the relationship with the owner, as measured through the Monash Dog-Owner Relationship (M/DORS).

Finally, two studies analysed sOT in relation to maternal behaviour in Labrador Retrievers. These studies measured sOT concentrations in dams prior to reunion with puppies for a period of 18 days of lactation (every 3 days from day 3 to 21). The first study showed an overall tendency for sOT to increase over time, and sOT concentration on the last day of data collection (day 21 of lactation) was significantly higher than on some of the previous days. In this study, maternal behaviours and mother-related factors were also analysed [39]. The other study focused on OT Receptor Gene Polymorphism and compared sOT levels and maternal behaviours in dams with different genotypes of this gene. sOT levels, as well as maternal behaviour, were correlated with a single nucleotide gene variant in the OT receptor [40].

The methods employed by these studies for sOT collection and analysis are shown in Table 2. None of the studies utilising ELISA kits mentioned extraction as a sample processing step. Furthermore, Table 2 lists the other measures considered in the examined studies in addition to sOT. In certain instances, these measures have been found to be associated with sOT or its variation over time, as for example behaviour [30,39], cortisol [32], percentage of male pups [39], or OT receptor gene polymorphism [40], but correlations between the collected variables were not sought or not present in every study.

The characteristics and size of the sample of the aforementioned studies were also considered. The sample size of the studies ranged from a minimum of one [35] to a maximum of 55 dogs [33]. Some studies involved specific dog breeds, such as French Bulldogs [38] or Labrador/Golden Retrievers [30,32,39,40], while others involved various breeds. The subjects were typically of varying ages, with some studies involving young adults [32] or mature adults [30,39]. The majority of the dogs were household pets, although some studies also targeted dogs from breeders [30,39,40], therapy dogs [34,35], or assistance dogs [30,32,33].

### 3.5. Studies Investigating Salivary Vasopressin (sAVP) in Dogs

In addition to the study by MacLean and colleagues [27] concerning the validation of sOT and sAVP analytical methods, which was previously discussed in Section 3.3, four studies investigated dogs’ sAVP. Two of these studies examined sAVP in relation to a positive human-animal interaction [30] or separation from the owner in dogs with separation-related problems (SRP) compared to control dogs [31]. In this latter study, dogs with SRP exhibited significantly elevated levels of sAVP in comparison to the control group following a three-minute separation from their owners. Furthermore, in the study by MacLean and colleagues [30], a significant increase in sAVP was observed in dogs exposed to the control condition, which, in contrast to the group exposed to affiliative HAI, provided that dogs were ignored by the experimenter for 10 min.

The remaining two studies investigated sAVP response to potential stressful stimuli. Specifically, Jeong and colleagues [41] analysed the dogs’ sAVP alongside vital parameters (body temperature, heart rate, respiratory rate, systolic blood pressure), behaviour, and serum cortisol prior to and following a car journey to the laboratory, a physical examination by a veterinarian, the sampling procedure, and a 30 min exposure to vacuum noise. The participant dogs were also divided into two groups based on their behavioural responses: those exhibiting signs of greater stress and those displaying signs of lesser stress. In the group exhibiting higher stress levels, sAVP exhibited a significant decrease following noise exposure and environmental stimulation and was negatively correlated with changes in blood pressure. In the study by Schroers and colleagues [42], two groups of dogs were treated with casozepine, a peptide with anxiolytic effects, or with a placebo, to assess stress during a veterinary examination and follow-up. The authors measured dog behaviour through a visual analogue scale, pulse rate, salivary cortisol, and owners’ perception of dog stress. The study revealed that sAVP concentrations increased significantly during follow-up only in the placebo group.

The methods employed for sAVP collection and analysis are outlined in Table 3. None of the studies performed a sample extraction phase. The sample size of the studies ranged from 26 to 42 dogs. With the exception of the study by MacLean and colleagues [30], which involved mature adult dogs from breeders or assistance dogs (Labrador or Labrador × Golden Retriever crosses), the other studies included pet dogs of various breeds and ages.

The detailed data charting for each study can be consulted in the Supplementary Material, comprising sAVP concentrations values when reported (see Supplementary Table S4).

## 4. Discussion

The measurement of OT and/or AVP in dog saliva is a relatively recent development, with all the included studies having been published within the last eight years. Consequently, it is challenging to derive or establish reliable reference values for sOT and sAVP in dogs. The methodological validation studies demonstrate the presence of considerable variability in the concentrations of the two analytes, which is partly contingent on the methods employed for collection and analysis [27,28,29]. This variability is further confirmed by the other studies included in this review, which showed that the average concentrations of sOT ranged from approximately 100 to over 3000 pg/mL, and the average concentrations of sAVP ranged from around 50 to 700 pg/mL. One hypothesis to explain this variability is that it reflects the difference in analysis methods employed. Nevertheless, this alone does not suffice to fully elucidate the phenomenon, as disparate studies employing similar analysis approaches still yield divergent ranges of values. Consequently, the observed variability may be indicative of disparities in experimental conditions or substantial inter-individual variability, as previously highlighted in the context of salivary cortisol [8,43]. The heterogeneity of characteristics among the samples from the included studies should be considered in this regard. Indeed, factors such as gender, reproductive status, age, and physiological conditions (e.g., lactation) are known to be associated with different individual endocrine assets, including the oxytocin-vasopressin system. To the authors’ knowledge, however, there are no large-scale studies that analyse the influence of these factors on OT and AVP concentrations in dogs. The genetic component has also been identified as a contributing factor to this variability, with evidence from the included studies demonstrating a correlation between OT receptor gene polymorphism, sOT concentrations, and maternal behaviour [40]. Furthermore, individual variations in oxytocin basal concentrations have been shown to be significantly heritable (i.e., under genetic control) in dogs and are linked to phenotypic diversification in aspects of temperament, behavioural laterality, and executive function [44]. Life experiences have also been demonstrated to have an influence on OT release during dog-human contact [45]. It is therefore highly probable that these genetic and epigenetic factors play a significant role in OT and AVP individual variability, as well as other factors. However, further research is required to determine their interplay, the relative importance of each factor, and the potential synergistic effects between factors. At the interspecific level, a further finding that emerges from the review is that concentrations of sOT in dogs exceed those observed in humans, probably due to the different proteomic profiles of saliva in the two species [27,36].

A series of key points can be discussed further on the basis of the analysed literature and other relevant literature on canine salivary bioscience. Firstly, the importance of uniform collection methods. The media used for saliva collection have been demonstrated to exert an effect on the determination of sOT and sAVP, as disparate physical properties of the swabs may have an influence on which components of saliva are absorbed [27]. Thus, despite the fact that different collection media appear to return correlated values in the same subject [27], this aspect must be borne in mind when comparing values from different studies and when reporting the method utilised, as it can also influence the study’s results. The majority of included studies utilise Salimetrics^®^ Children’s swabs, which have been validated for multiple analytes and are of practical use [27]. However, four alternative devices are documented in the analysed literature, in particular the Sarstedt Salivette^®^. Other salient aspects related to saliva collection include the duration of collection and the utilisation of salivary stimulants. In most studies, the sponge is inserted into the dog’s mouth, typically in the cheek, for approximately one minute, without using salivary stimulants. Indeed, salivary stimulants such as citric acid or food residues resulted in interference with the subsequent detection of both sOT and sAVP [27], as already reported in the case of salivary cortisol [43]. For the same reason, food, water, and other elements that could interfere with salivary analyte concentration should not be provided before salivary collection [46]. The simulation of salivation by the odour of food treats, as used in two studies among those included in this review [31,33], could be a good alternative. Two studies reported the necessity of repeating the sampling process when the initial volume was inadequate [33] or when there was insufficient saliva for the measurement of multiple analytes [36]. This could limit the feasibility of using salivary-based assays in clinical settings, where it would be impossible to repeat the sampling procedure. In the design of any study that incorporates this collection method, moreover, it is imperative to acknowledge that not all animals possess the necessary acclimatisation to the use of salivary tampons. This can result in instances of discomfort or reluctance to retain the tampon within the oral cavity for the stipulated duration, thereby leading to inadequate salivary volumes, or even, in some cases, a risk of safety for the researcher [47]. This aspect must be considered when planning procedures that are to be carried out with minimal invasiveness and respect for animal welfare. Salivary collection is a less invasive procedure than blood extraction, which is a significant advantage. However, it is necessary that dogs are used to the manipulation or properly trained, as in the study by Akiyama and Ohta [36], in which owners were asked to brush their dogs’ teeth before the experiment to accustom them to the manipulation of the mouth. It is strongly recommended that some form of habituation, or positive training to the collection procedure, be implemented to ensure the well-being of the animal and to avoid any influence by the collection procedures on the results.

Secondly, the issue of the optimal timing for sample collection constitutes a significant point of concern in the investigation of sOT and sAVP. The precise temporal peak of sOT and sAVP, as well as the mechanisms by which these hormones reach saliva in dogs, are not fully understood [27]. In a pilot experiment conducted as part of the study by MacLean and colleagues [30], the authors sought to ascertain the most suitable sampling periods for detecting changes in sOT and sAVP within a 10 min HAI testing paradigm. This entailed the measurement of oxytocin concentration prior to and five and ten minutes following the interaction. In this instance, a significant increase in sOT was observed 10 min after the interaction. Moreover, in the studies included in this review, salivary samples were frequently collected immediately after the condition/stimulus (ranging from 3 to 30 min in duration). Nevertheless, this is also a fundamental aspect to further investigate through pharmacokinetics studies, as the timing of OT and AVP distribution and metabolism has the potential to influence the overall results of the studies and their interpretation.

A number of other considerations can be raised in relation to the analytical methods. Some of these aspects have also been presented extensively by López-Arjona and colleagues [48] in a review on sOT analysis in different species, including humans. In relation to the processing of the samples, the two studies that employed different extraction techniques concluded that this step is not required [27,29], and it was not reported in any of the other studies included in this review. The ELISA kits in the examined literature are those from Cayman, Arbor, Enzo Life Science for sOT, or Enzo Life Science, MYbiosource, NordicBioSite for sAVP. However, the validation studies comparing different analytical methods have documented the absence, or inadequacy, of correlations between disparate commercially available methods in the context of dog saliva, possibly because they measure different oxytocin forms [27,29]. For instance, in the study by López-Arjona and colleagues [29], the significance of the results changed when monoclonal or polyclonal antibodies were used. The authors hypothesised that the sOT forms measured by the monoclonal assay are more involved in stroking than other sOT forms and would be more related to triggering positive emotions in the dog. Given the impact of analytical methods on the resulting sOT and sAVP concentrations, the standardisation of analytical methods would be an ideal step for conducting future studies. The present review of the literature suggests that the majority of authors have not utilised extraction techniques and that ELISA kits are more frequently employed for the analysis. In any case, it is essential to describe thoroughly the preparation and analysis of samples, and the utilised assays. The issue of which forms of sOT are more significant for the different experimental paradigms and the most appropriate way of analysing them remains to be elucidated. A range of further challenges have emerged in the course of this review, in line with those previously identified by other authors. These issues pertain to the difficulty of utilising and interpreting sOT and sAVP, given the multitude of their functions, forms and the lack of knowledge regarding the correspondence between central and peripheral peptide release [11,16,18,49,50]. For example, in the context of human-animal interaction, the included studies, which employed experimental procedures in which dogs were exposed to positive affiliation stimuli, resulted in an increase in sOT [29,30,32]. In contrast, negative conditions (i.e., isolation) appear to have no impact on sOT concentrations [31,32] as well as more naturalistic, complex and protracted social interactions such as AAIs or walking with owner [36]. Further investigation is therefore necessary to determine the kind and frequency of stimulation that induces OT release in dogs and detectable changes in sOT levels. From the included studies, an increase in sOT is evident in those employing a short testing paradigm involving exposure to standardised positive stimuli, such as human-animal contact. These study designs would also be easier to replicate in order to gain a better understanding of the dynamics of sOT fluctuation. It has been demonstrated that physical contact, such as stroking and petting the dog, plays a significant role in the release of this hormone [51,52]. However, given OTs stress-buffering function, it has been demonstrated that stress-inducing stimuli can also induce OT release [53]. Furthermore, the study by López-Arjona and colleagues [29] showed that groups of dogs with different characteristics responded differently to an allegedly positive stimulus. It is acknowledged that emotions are not merely a response to external stimuli; they are also a product of subjective interpretation and influenced by factors such as personality and past experiences [2,3]. Similarly, a recent study by Scandurra and colleagues (2024) concluded that active engagement with humans is imperative for the benefits of social buffering in both goats and dogs, based on cortisol measurements. The study emphasised the significance of considering individual factors linked to experience, such as acceptance of social interaction, in understanding the impact of social buffering [54]. Consequently, it is necessary to acknowledge the subjective component of emotions when assessing their impact and function in various contexts, including hormonal responses. Similarly, sAVP was found to increase in response to a ten-minute isolation [30] and to be higher in dogs with separation-related problems than control dogs [31], however, in the study by Jeong and colleagues (2020), a more prolonged exposure to environmental stress stimuli led to its decrease. The authors of the study posit that this phenomenon could be attributed to the role of AVP in blood pressure regulation, functioning as an anti-diuretic hormone, which involves a negative feedback loop. Consequently, stress-induced elevation in blood pressure may have led to the inhibition of AVP release. Given the multifaceted homeostatic mechanisms implicated, it is challenging to draw definitive conclusions regarding the variations exhibited by sAVP, particularly in response to prolonged and diversified stimuli. In summary, the function of both hormones is contingent on various social contexts and individual factors, the specific site of action in the brain, the distribution and state of their receptors, and other related factors [16,29,41]. This complexity appears to complicate their use as welfare indicators, particularly in naturalistic or semi-naturalistic experimental settings where dogs are exposed to a variety of stimuli. Furthermore, as previously mentioned, there is a documented divergence between study results comparing central and peripheral measurements of the concentrations of these hormones [48]. This phenomenon, which requires further elucidation, poses a risk of leading to erroneous conclusions when measuring OT and AVP from blood or saliva, as opposed to cerebrospinal fluid (which is considerably more invasive). Inferences regarding central effects based on peripheral measurements should thus be made with caution, at least until the effective correlation between different samples has been clarified. Finally, the included studies employed a variety of measures in conjunction with sOT and sAVP, encompassing behavioural and physiological parameters (e.g., cortisol, alpha-amylase, heart rate, and heart rate variability). However, the observed associations between these parameters were not consistently present or in accordance with expectations, as for instance in Hill and colleagues [35]. Given the context dependency and individual variability in OT and AVP responses, future studies should prioritise the systematic correlation of hormonal changes with standardised behavioural measures. This integrative approach would enhance the interpretability of physiological data and strengthen the validation of sOT and sAVP as reliable welfare indicators in dogs. Moreover, as previously mentioned, it is known that the oxytocin-vasopressin system interacts by complex feedback loops with various body systems, including, to name a few, the hypothalamic-pituitary-adrenal (HPA) and sympathoadrenal axis, the immune system and the dopaminergic system [10,11,55]. Therefore, multiparametric assessments of animal welfare are preferred over relying on a single parameter, although the understanding of how to evaluate different measures in combination to obtain a good representation of an animal’s emotional state remains to be elucidated [10]. Going even further, given that OT and AVP are both linked to the social sphere, another area of investigation is the interaction of these systems between individuals, as has been studied in the case of OT between dogs and their owners [56].

The limitations of this review can be attributed to the exclusion criteria applied to the literature. These criteria excluded papers in languages other than English and conference proceedings, with the possibility of losing relevant data. In addition, a more comprehensive raw data collection process from the authors would have enabled a meta-analysis as outlined by Cobb and colleagues [8]. However, due to limitations in time and resources, further data collection and analysis were not performed.

## 5. Conclusions

This scoping review identified 16 recent studies that measured sOT and/or sAVP in dogs. This facilitated the identification of methodological and interpretive considerations related to these two neuropeptides, which play a key role in the processes of bonding and social attachment across numerous species. Furthermore, it enabled the mapping of the research contexts in which these indicators have been employed, including their intended purpose and the challenges associated with their use. As recently discussed by Cobb and colleagues [10], the increasingly evolving scientific understanding of physiological indicators of dog welfare raises concerns about their construct validity, and further investigation and a multidimensional approach to canine welfare assessment are needed.

## Figures and Tables

**Table 1:** Methodological differences in the three development and validation studies for dog’s sOT and sAVP. ELISA: Enzyme-Linked Immunosorbent Assay; HPLC-MS: High-Performance Liquid Chromatography-Mass Spectrometry; LC-MS: liquid chromatography-mass spectrometry; sAVP: salivary vasopressin; sOT: salivary oxytocin. * Some studies employed more than one method.

Methods	Details	Number of Studies *	Reference
**Saliva collection media**	Salimetrics^®^ Children’s swab (Salimetrics LLC, State College, PA, USA)	1	[27]
	Sarstedt Salivette^®^ (Sarstedt AG&Co., Nümbrecht, Germany)	1	[27]
	VERSISAL^®^ kit Oasis Diagnostics (Vancouver, WA, USA)	1	[28]
	Sarstedt Salivette^®^ tube containing a sponge (Esponja Marina, La Griega E. Koronis, Madrid, Spain)	1	[29]
**Salivation stimuli before collection**	No/not reported	3	[27,28,29]
	Kibble	1	[27]
	Citric acid solution	1	[27]
**Duration of collection**	1 min	2	[27,29]
	2 min or more	1	[27,28]
**Sample processing**	Extraction	2	[27,28]
	No extraction or reduction/alkylation	2	[27,29]
	Reduction/alkylation	1	[29]
**Analysis method/kit sOT**	ELISA kit from Cayman Chemical (Ann Arbor, MI, USA)	2	[27,29]
	ELISA kit from Arbor Assays (Ann Arbor, MI, USA)	1	[27]
	ELISA Enzo Life Sciences (pilot, human sOT) (Farmingdale, NY, USA)	1	[27]
	HPLC-MS	1	[27]
	LC-MS	1	[28]
	AlphaLISA with monoclonal antibody	1	[29]
	AlphaLISA with polyclonal antibody	1	[29]
**Analysis method/kit sAVP**	ELISA Enzo Life Sciences (Farmingdale, NY, USA)	1	[27]
	HPLC-MS	1	[27]

**Table 2:** Methodological differences in sOT collection and analysis (12 studies). ELISA: Enzyme-Linked Immunosorbent Assay; LC-MS: liquid chromatography-mass spectrometry; N: number of studies; sAVP: salivary vasopressin; sOT: salivary oxytocin; UHPLC: Ultra-High Performance Liquid Chromatography. * Some studies can employ multiple measures.

Methods	Details	N.	Reference
**Saliva collection media**	Salimetrics^®^ Children’s swab (Salimetrics LLC, State College, PA, USA)	5	[30,31,33,35,37]
	Sarstedt Salivette^®^ (Sarstedt AG&Co, Nümbrecht, Germany)	4	[32,38,39,40]
	Sarstedt Salivette^®^ containing a sponge	1	[29]
	VERSISAL^®^ Oasis Diagnostic (Vancouver, WA, USA)	1	[34]
	Mentip^®^ hospital cotton swab (JCB Industry Limited, Tokyo, Japan)	1	[36]
**Salivation stimuli**	No/not mentioned	10	[29,30,32,34,35,36,37,38,39,40]
	Odour of food	2	[31,33]
**Duration of collection**	1 min	8	[29,30,31,32,36,38,39,40]
	1 to 2 min	2	[35,37]
	2 min or more	2	[33,34]
**Analysis method/kit sOT**	ELISA kit from Cayman Chemical	6	[30,32,35,37,39,40]
	ELISA kit from Arbor Assay	2	[31,33]
	ELISA kit from Enzo Life Sciences (Farmingdale, NY, USA)	2	[36,38]
	LC-MS with UHPLC	1	[34]
	AlphaLISA with monoclonal and polyclonal antibody	1	[29]
Other measures *	sAVP	2	[30,31]
	Salivary cortisol	4	[32,34,35,36]
	Salivary alpha-amylase	1	[35]
	Plasma OT	1	[30]
	Plasma AVP	1	[30]
	Urinary OT	1	[33]
	Tympanic membrane temperature	1	[34]
	Heart rate and heart rate variability	1	[34]
	Behaviour	10	[29,30,31,32,33,35,37,38,39,40]
	Monoamines	1	[36]
	GABA	1	[36]
	Demographic and questionnaire data	2	[33,38]
	Mother-related factors	1	[39]
	OT receptor gene polymorphism	1	[40]

**Table 3:** Methodological differences in sAVP collection and analysis (4 studies). ELISA: Enzyme-Linked Immunosorbent Assay; N: number of studies.

Methods	Details	N.	Reference
**Saliva collection media**	Salimetrics^®^ swab (Salimetrics LLC, State College, PA, USA)	4	[30,31,41,42]
**Salivation stimuli**	No/not mentioned	3	[30,41,42]
	Food odour	1	[31]
**Duration of collection**	1 min	3	[30,31,42]
	1–3 min	1	[41]
**Analysis method/kit sAVP**	ELISA Enzo Life Sciences (Farmingdale, NY, USA)	2	[30,41]
	ELISA MyBioSource (San Diego, CA, USA)	1	[31]
	ELISA NordicBioSite (Täby, Sweden)	1	[42]

## Data Availability

Data are contained within the article or Supplementary Material.

## Supplementary Materials

The following supporting information can be downloaded at: https://www.mdpi.com/article/10.3390/ani15162421/s1, Table S1: Data charting of the development and validation studies for salivary oxytocin (sOT) analysis in dogs (n = 3); Table S2: Data charting of the development and validation studies for salivary vasopressin (sAVP) analysis in dogs (n = 1); Table S3: Data charting of the experimental studies measuring salivary oxytocin (sOT) in dogs (n = 12); Table S4: Data charting of the experimental studies measuring salivary oxytocin (sAVP) in dogs (n = 4).

## Abbreviations

The following abbreviations are used in this manuscript:

HPA	Hypothalamic-Pituitary-Adrenal
OT	Oxytocin
AVP	Vasopressin
sOT	Salivary Oxytocin
sAVP	Salivary Vasopressin
PRISMA-ScR	Preferred Reporting Items for Systematic Reviews and Meta-Analyses extension for Scoping Reviews
WoS	Web of Science
ELISA	Enzyme-Linked Immunosorbent Assay;
HPLC-MS	High-Performance Liquid Chromatography-Mass Spectrometry
LC-MS	Liquid chromatography-mass spectrometry
HAI	Human-animal interaction
AAIs	Animal-assisted interventions
M/DORS	Monash Dog-Owner Relationship
UHPLC	Ultra-High Performance Liquid Chromatography
SRP	Separation-Related Problems
