# Supplementary material for: A Scoping Review on Salivary Oxytocin and Vasopressin Measurement in the Dog
Source: Animals (Basel). 2025 Aug 18;15(16):2421. doi: 10.3390/ani15162421 (PMC12383078; doi:10.3390/ani15162421)
Supplement: Supplementary file 1 [file animals-15-02421-s001.zip › animals-3700862-supplementary.pdf]

Supplementary Table S1. Data charting of the development and validation studies for salivary oxytocin (sOT) analysis in dogs (n=3). ELISA: Enzyme-Linked Immunosorbent Assay; HPLC-MS: High-Performance Liquid Chromatography-Mass Spectrometry; LC-MS: liquid chromatography-mass spectrometry; R/A: reduction/alkylation; min: minutes; sAVP: salivary vasopressin.

First author (Year): Title	Objective of the study	Collection methods	Collection duration	Timepoint/condition	sOT Processing/Analysis method/Assay type	sOT Concentration
McLean et al. (2018): Validation of salivary oxytocin and vasopressin as biomarkers in domestic dogs	Methodological validation of sOT and sAVP analysis comparing different sample collection techniques, processing procedures and analysis methods	Salimetrics® Children's swab or Sarstedt Salivette® with different stimuli for salivation (kibble, solution of citric acid, none)	1 min	\	ELISA Cayman[1]/non-extracted	median = 679 pg/mL (range = 356–1073 pg/mL)
					ELISA Arbor[2]/non-extracted	median = 258 pg/mL (range = 207–471 pg/mL)
					ELISA Enzo[3] OT/non-extracted	mean = 690 pg/mL
					ELISA Cayman[1]/extracted	median = 260 pg/mL (range = 181–418 pg/mL)
					ELISA kit Arbor[2]/extracted	median = 41 pg/mL (range = 27–105 pg/mL)
					HPLC–MS	median = 18 pg/mL (range = 8–49 pg/mL)[4]
	Physiological validation of sOT analysis through comparison with plasma oxytocin released in association with milk let-down	Salimetrics® Children's swab	2.5 min (range 2-4)	T0 (before)	ELISA non-extracted	mean = 626.7 pg/mL (range = 475.5–871.0 pg/mL)
				T1 (during nursing)		mean = 1051.7 pg/mL (range = 564.9–1448.1 pg/mL)
				T2 (after 10 min nursing)		\
Wang et al. (2019): Development and validation of a simple LC-MS method for the quantification of oxytocin in dog saliva	Develop and validate a LC-MS analytical method for the quantification of dog sOT, and use LC-MS method to evaluate dog sOT concentrations from multiple collections.	VERSISAL® kits Oasis Diagnostics	2 min	\	LC-MS/extracted	reported graphically

[1] ELISA kit from Cayman Chemical.

[2] ELISA kit from Arbor Assays.

[3] ELISA kit from Enzo Life Sciences – pilot analysis.

[4] 9 on 20 samples below the limit of detection (~2 pg/mL).

Lopez-Arjona et al. (2021): Changes in salivary oxytocin after stroking in dogs: validation of two assays for its assessment[5]	Evaluation and analytical validation of two new methods for the measurement of dog sOT (monoclonal and polyclonal assay)	Sarstedt Salivette® tube containing a sponge (Esponja Marina, La Griega E. Koronis)	1 min	\	AlphaLISA monoclonal with R/A	median = 303.3 pg/mL; 25-75th percentile: 190.2–371.1 pg/mL
					AlphaLISA monoclonal without R/A	median = 301.5 pg/mL; 25-75th percentile: 167.7–424.3 pg/mL
					AlphaLISA polyclonal with R/A	median = 12.8 ng/mL; 25-75th percentile: 8.5–19.2 ng/mL
					AlphaLISA polyclonal without R/A	median = 25.2 ng/mL; 25-75th percentile: 11.4–71.8 ng/mL
					ELISA Cayman[6]/with R/A	median = 208.8 pg/mL; 25-75th percentile: 155.7–257.4 pg/mL
					ELISA Cayman[6]/without R/A	median = 58.4 pg/mL; 25-75th percentile: 43.4–95.4 pg/mL

[5] This study also performed an experimental trial that is reported in Supplementary Table 3.
[6] ELISA kit from Cayman Chemical.

Supplementary Table S2. Data charting of the development and validation studies for salivary vasopressin (sAVP) analysis in dogs (n=1). ELISA: Enzyme-Linked Immunosorbent Assay; HPLC-MS: High-Performance Liquid Chromatography-Mass Spectrometry; min: minutes; sOT: salivary vasopressin.

First author (Year): Title	Objective of the study	Collection methods	Collection duration	Timepoint/condition	sAVP Processing/Analysis method/Assay type	sAVP Concentration
McLean et al. (2018): Validation of salivary oxytocin and vasopressin as biomarkers in domestic dogs	Methodological validation of sOT and sAVP analysis comparing different sample collection techniques, processing procedures and analysis methods	Salimetrics® Children's swab or Sarstedt Salivette® with different stimuli for salivation (kibble, solution of citric acid, none)	1 min	\	ELISA Enzo[7] AVP/non-extracted	median = 454 pg/mL (range = 228–1489 pg/mL)
					ELISA Enzo[7] AVP/extracted	median = 5 pg/mL (range = 2–11 pg/mL)[8]
					HPLC–MS	median = 25 pg/mL (range = 5–73 pg/mL)[9]

[7] ELISA kit from Enzo Life Sciences.
[8] Values near the limit of detection of the kit.
[9] 5 on 20 samples below the limit of detection (~2 pg/mL).

Supplementary Table S3. Data charting of the experimental studies measuring salivary oxytocin (sOT) in dogs (n=12). HAI: human-animal interaction; ELISA: Enzyme-Linked Immunosorbent Assay; UHPLC: Ultra-High Performance Liquid Chromatography; LC-MS: liquid chromatography-mass spectrometry; min: minutes; NA: not applicable; NR: not reported; sAVP: salivary vasopressin; SRP: Separation-related problems; HR: heart rate; HRV: heart rate variability.

First author (Year): Title	Objective	SALIVARY COLLECTION			SAMPLE				EXPERIMENTAL DESIGN			OT Analysis	OT Concentration (pg/mL)	Other measures
		Collection media	Stimuli	Min	Size	Breed	Age class[10]	Dog Type	Group (size)	Condition	Timepoint			
McLean et al. (2017): Effects of affiliative human–animal interaction on dog salivary and plasma oxytocin and vasopressin	Pilot study to identify the optimal sampling periods for detecting changes in sOT and sAVP	Salimetrics®	No	1	10	Labrador, retriever crosses	Adolescent	Breeder	NA	10 min HAI (experimenter)	T0 (before)	ELISA Cayman	NR	sAVP, plasma OT and AVP
											T1 (after 5 minutes HAI)		NR	
											T2 (after 10 minutes HAI)		NR	
	Compare OT and AVP in plasma and saliva in HAI versus control group in association with behaviour	Salimetrics®			38	Labrador, retriever crosses	Adolescent/Mature adult	Assistance/guide	Experimental (19)	10 min HAI (experimenter)	T0 (before)	ELISA Cayman	mean = 630	Behaviour, sAVP, plasma OT and AVP
											T1 (immediately after)		mean = 874	
									Control (19)	no HAI	T0 (before)		mean = 563	
											T1 (immediately after)		mean = 642	
Pirrone et al. (2019): Salivary vasopressin as a potential non–invasive biomarker of anxiety in dogs diagnosed with separation–related problems	Examine the behaviour and OT and AVP fluctuations in dogs in response to a human-dog interaction that might potentially induce anxiety in dogs	Salimetrics®	Food odour	1	26	Various	Various	Pet	Dogs with SRP (13)	3 min separation[11]	T0 (before)	ELISA Arbor	median = 127.87	sAVP and behaviour
											T1 (immediately after reunion)		median = 138.79	
											T2 (10 minutes after reunion)		median = 67.04	
									Control dogs (13)		T0 (before)		median = 149.99	
											T1 (immediately after reunion)		median = 183	
											T2 (10 minutes after reunion)		median = 221.60	
Ogi et al. (2020): Effects of stroking on salivary oxytocin and	Investigate how dog sOT levels are affected by human interaction and isolation	Sarstedt	No	1	8	Labrador and golden retriever	Adolescent	Assistance/guide	NA	5 min positive condition (affiliative interactio	T0 (before)	ELISA Cayman	median = 172.77 (117.97–282.23)	Salivary cortisol and behaviour
											T1 (immediately after)		median = 193.77 (135.80–433.11)	

[10] The categorisation of age classes is as follows: puppy (less than 6 months); adolescent (6-24 months); mature adult (2-6 years); senior (7-11 years); geriatric (older than 12 years). The relevant details for the classification are reported in the Methods section. The term 'Various' was utilised in instances where the reported age of the sample fell within three or more age classes.

[11] Simplified version of the Ainsworth's strange situation test.

cortisol in guide dogs: preliminary results										n with trainer)				
										5 min negative condition (isolation)	T0 (before)		median = 193.97 (111.10–372.10)	
											T1 (immediately after)		median = 211.35 (105.30–374.10)	
Clark et al. (2020): Physiological state of therapy dogs during animal-assisted activities in an outpatient setting	Evaluate the emotional state of therapy dogs in response to animal assisted activities in an outpatient clinical setting	VERSISAL®	No	2	19	Various	Various	Therapy	NA	Animal assisted activity with patients with fibromyalgia (20 min session)	T0 (before) - 18 dogs, repeated measures (76)	LC–MS with UHPLC	mean = 1007.19 (90.65-2366.9)	HR, HRV, tympanic temperature, salivary cortisol
											T1 (after) - 8 dogs, repeated measures (76)		mean = 1007.19 (261.87-2427.33)	
Akiyama et al. (2021): Hormonal and neurological aspects of dog walking for dog owners and pet dogs	Investigate the effects of dog walking on both owners and dogs, focusing on sOT and cortisol	Mentip® cotton swab	No	1	10	Golden, Labrador, Shiba	Various	Pet	walk with dog	30 min walk	T0 (before)	ELISA Enzo OT	mean = 275.0, SE 25.1	Salivary cortisol[12]
											T1 (15 minutes walk)		mean = 329.3, SE 26.9	
											T2 (30 minutes walk)		mean = 312.9, SE 21.8	
											T3 (10 minutes after the end)		mean = 325.3, SE 27.8	
									walk without the dog		NA	NA	NA	
	Investigate the effects of dog walking on both owners and dogs, focusing on brain neural activity				14	Various	Mature adult/Senior	Pet	walk with dog	30 min walk	T0 (before)	ELISA Enzo OT	mean = 284.5, SE 19.0	Salivary cortisol, monoamine (including metabolites), and GABA (gamma-
											T1 (15 minutes walk)		mean = 332.7, SE 20.9	
											T2 (30 minutes walk)		mean = 345.0, SE 25.2	
											T3 (10 minutes after the end)		mean = 312.2, SE 28.9	
									walk without the dog		NA	NA	NA	

[12] Measures taken also from the owner.

														aminobutyric acid)[12,13]
Ogi et al. (2021): Oxytocin receptor gene polymorphism in lactating dogs	Investigate the possible correlation between OT receptor gene polymorphism, peripheral sOT and maternal behaviour in dogs.	Sarstedt	No	1	19	Labrador	Mature adult	Breeder	AA genotype (2)<br>AT genotype (5)<br>TT genotype (12)	Lactation	Repeated measures every 3 days from day 3 to 21. Before reunion with the puppies.	ELISA Cayman	Reported graphically	Behaviour, single nucleotide polymorphisms genotyping
Lopez-Arjona et al. (2021): Changes in salivary oxytocin after stroking in dogs: validation of two assays for its assessment	Experimental trial in which dogs were stroked by their owners and analysis through monoclonal and polyclonal assays	Sarstedt with sponge	No	1	17	Various	NR	Pet	Group 1 (8)[14]	10 min stroking (owner)	TB (basal)	AlphaLISA monoclonal	median = 335.5; Q1: 270.4–Q3: 1564.0	Dog behaviour evaluated by the owner
											T0 (immediately after)		median = 607.7; Q1: 442.0–Q3: 1786.0	
											T15 (15 min after)		median = 904.3; Q1: 459.7–Q3:3143.0	
									Group 2 (9)[15]		TB (basal)		median = 993.4; Q1: 461.7–Q3: 2372.0	
											T0 (immediately after)		median = 582.4; Q1: 441.3–Q3: 1003.0	
											T15 (15 min after)		median = 452.7; Q1: 345.0–Q3: 806.2	
									Group 1 (8) [14]		TB (basal)<br>T0 (immediately after)<br>T15 (15 min after)	AlphaLISA poly	Reported graphically (ng/mL)	
											TB (basal)			

[13] Assays of saliva samples were performed in the order of oxytocin, cortisol, monoamine (including metabolites), and GABA. If the sample amount was not sufficient (400 μL or less), the measurement had to be omitted in the reverse order. Therefore, the samples of the dogs' measuring GABA were only 20.
[14] Dogs exhibiting more acceptance of the sponge and relaxation during stroking.
[15] Dogs exhibiting less acceptance of the sponge and relaxation during stroking.

									Group 2 (9)[15]		T0 (immediately after)<br>T15 (15 min after)	clon al		
Ogi et al. (2021): The influence of oxytocin on maternal care in lactating dogs	Investigate if the sOT was associated with maternal care provided to the offspring in lactating dams	Starstedt	No	1	25	Labrador	Mature adult	Breed er	NA	Lactation	7 samples (every 3 days from day 3 to 21). Before reunion with the puppies.	ELIS A Cay man	Reported graphically	Behaviour and mother related factors
Hill et al. (2023): Investigating dog welfare when interacting with autistic children within canine-assisted occupational therapy sessions: a single case study	Ascertain if participation within canine-assisted therapy sessions with children with autism spectrum disorder produced elevated stress levels for a therapy dog, as measured by significant differences in behavioural observations, salivary cortisol, alpha amylase, and OT, when compared to days spent unemployed at home	Salimetric s®	No	1,5	1	Labradoo dle	Mature adult	Thera py	NA	Days at home (baseline)	Wk 1<br>Wk 4<br>Wk 7	ELIS A Cay man	mean = 995.8<br>mean = 1118.7<br>mean = 681.9	Behaviour and salivary cortisol and alpha amylase
										Treatmen t days: therapy session with children with autism (average 3 collection days)	Wk 1 Pre first<br>Wk 1 Mid first<br>Wk 1 Post first<br>Wk 1 Pre last<br>Wk 1 Mid last<br>Wk 1 Post last<br>Wk 4 Pre first<br>Wk 4 Mid first<br>Wk 4 Post first<br>Wk 4 Pre last<br>Wk 4 Mid last<br>Wk 4 Post last<br>Wk 7 Pre first<br>Wk 7 Mid first<br>Wk 7 Post first<br>Wk 7 Pre last<br>Wk 7 Mid last<br>Wk 7 Post last		mean = 1397.7<br>mean = 2151.2<br>mean = 1118.7<br>mean = 2309.9<br>mean = 1835.7<br>mean = 2018.1<br>mean = 443.3<br>mean = 316.7<br>mean = 271.6<br>mean = 1189.5<br>mean = 823.8<br>mean = 325.0<br>mean = 1442.6<br>mean = 804.0<br>mean = 996.6<br>mean = 2161.4<br>mean = 355.5<br>mean = 1485.9	
McGetrick et al. (2024): Do pet dogs reciprocate the receipt of food from familiar and unfamiliar conspecifics?	1) Determine whether pet dogs would reciprocate the receipt of food from other pet dogs; 2) Investigate the effect of familiarity on reciprocity; 3) Determine whether changes in sOT were	Salimetric s®	No	1-2	24	Various	Various	Pet	Familiar dog test (12)	helpful	T0 (before)<br>T1 (after)	ELIS A Cay man	Reported graphically	Behaviour (button presses)
										unhelpful	T0 (before)<br>T1 (after)			
									Unfamil iar dog test (12)	helpful	T0 (before)<br>T1 (after)			
										unhelpful	T0 (before)<br>T1 (after)			

	associated with the experience of a cooperative or non-cooperative act and predictive of later reciprocation													
Gnanadesikan et al. (2024): Effects of human-animal interaction on salivary and urinary oxytocin in children and dogs	Explore the effects of naturalistic interactions between children and dogs on sOT release in both species and associations between methylation of the oxytocin receptor gene (OXTRm) and social behaviour	Salimetrics®	Food odour	2 [16]	55	Various	Mature adult	Pet	Familiar dog (54)	Interaction with a child	T1 (before)	ELISA Arbor	median = 217.41	Urinary OT, surveys, behaviour (of the dyad)[17]
											T2 (after 15 min interaction)		median = 253.33	
						Labrador	Senior	Assistance/guide	Unfamiliar dog (1)		T1 (before)		median = 317.11	
											T2 (after 15 min interaction)		median = 268.47	
Peterca et al. (2024): Dog-owner relationship and its association with social cognition in French Bulldogs	Investigate the relationship between social cognition, the dog–owner relationship and sOT	Starstedt	No	0,75-1	26	French Bulldogs	Various	Pet	NA	Two-way object test	15 min after arrival	ELISA Enzo OT	mean = 87.07 ± 53.11 (range = 15.6-217)	Demographic data, Monash Dog Owner Relationship Scale (MDORS), behaviour

[16] Repeated 1 more minute depending on the salivary volume collected.
[17] Also collected: children's salivary and urinary OT, methylation of the OT receptor gene in children.

Supplementary Table S4. Data charting of the experimental studies measuring salivary vasopressin (sAVP) in dogs (n=4). HAI: human-animal interaction; ELISA: Enzyme-Linked Immunosorbent Assay; min: minutes; sOT: salivary oxytocin; SRP: Separation-related problems; BT: body temperature; HR: heart rate; RR: respiratory rate; BP: blood pressure; VAS: visual analog scale.

		SALIVARY COLLECTION			SAMPLE				EXPERIMENTAL DESIGN					
**First author (Year): Title**	**Objective**	**Collection media**	**Stimuli**	**Min**	**Size**	**Breed**	**Age class[18]**	**Dog Type**	**Group (size)**	**Condition**	**Timepoint**	**AVP Analysis**	**AVP Concentration (pg/mL)**	**Other measures**
MacLean et al. (2017): Effects of affiliative human–animal interaction on dog salivary and plasma oxytocin and vasopressin	Pilot study to identify the optimal sampling periods for detecting changes in sOT and sAVP	Salimtrics®	No	1	10	Labrador, retriever crosses	Adolescent	Breeder	NA	10 min HAI (experimenter)	T0 (before)<br>T1 (after 5 minutes HAI)<br>T2 (after 10 minutes HAI)	ELISA Enzo AVP	NR<br>NR<br>NR	sOT, plasma OT and AVP
	Compare OT and AVP in plasma and saliva in HAI versus control group in association with behaviour	Salimetrics®			38	Labrador, retriever crosses	Adolescent/Mature adult	Assistance/guide	Experimental (19)	10 min HAI (experimenter)	T0 (before)<br>T1 (immediately after)	ELISA Enzo AVP	mean = 605<br>mean = 546	sOT, behaviour, plasma OT and AVP
									Control (19)	no HAI	T0 (before)<br>T1 (immediately after)		mean = 546<br>mean = 724	
Pirrone et al. (2019): Salivary vasopressin as a potential non–invasive biomarker of anxiety in dogs diagnosed with separation–related problems	Examine behaviour, OT and AVP in dogs in response to a HAI that might induce anxiety in dogs	Salimetrics®	Food odour	1	26	Various	Various	Pet	Dogs with SRP (13)	3 min separation[19]	T0 (before)<br>T1 (after reunion)<br>T2 (10 min after)	ELISA MyBioSource	median = 105.97<br>median= 136.61<br>median = 114	sOT, behaviour
									Control dogs (13)		T0 (before)<br>T1 (after reunion)<br>T2 (10 min after)		median = 90.40<br>median = 80.12<br>median= 81.53	
Jeong et al. (2020): Evaluation of salivary vasopressin as an acute stress biomarker in healthy dogs with stress	Evaluate the possibility of using sAVP as an acute stress biomarker in dogs	Salimetrics®	No	1-3	28	Various	Various	Pet	<stressed (14)	30 min Environmental stressful stimuli	T0 (before)<br>T1 (after)	ELISA Enzo AVP	mean = 82.61 ± 68.77<br>mean = 65.81 ± 53.50	BT, HR, RR, non-invasive systolic BP, serum
									>stressed (14)		T0 (before)<br>T1 (after)		mean = 78.07 ± 25.87<br>mean = 63.69 ± 20.16	
											T0 (before)		mean = 80.35 ± 55.46	

[18] The categorisation of age classes is as follows: puppy (less than 6 months); adolescent (6-24 months); mature adult (2-6 years); senior (7-11 years); geriatric (older than 12 years). The relevant details for the classification are reported in the Methods section. The term 'Various' was utilised in instances where the reported age of the sample fell within three or more age classes.
[19] Simplified version of the Ainsworth's strange situation test.

due to noise and environmental challenges									Total (28)		T1 (after)		mean = 65.00 ± 43.14	cortisol, behaviour
Schroers et al. (2024): Effect of casozepine administration on stress in dogs during a veterinary examination - A randomized placebo-controlled trial	Investigate the stress-reducing effect of a casozepine before a veterinary examination in dogs	Salimetrics®	No	1	42	NR	Various	Pet	Casozepine (22)	10 min Veterinary examination	Day 1 - before<br>Day 1 - after<br>Day 3 - before<br>Day 3 - after	ELISA NordicBioSite	Reported graphically	stress score, pulse rate, salivary cortisol, owner's perception
							Various		Placebo (20)		Day 1 - before<br>Day 1 - after<br>Day 3 - before<br>Day 3 - after			
